# Evaluation of the Marginal Fit of Computer-Aided Design (CAD) and Computer-Aided Manufacturing (CAM) Zirconia Bridges Fabricated With Different Firing Cycles of Veneering Porcelain: An In Vitro Study

**DOI:** 10.7759/cureus.34842

**Published:** 2023-02-10

**Authors:** Hawraa Chokr, Fendi Alshaarani, Hassan A Husein

**Affiliations:** 1 Department of Fixed Prosthodontics, Faculty of Dental Medicine, Damascus University, Damascus, SYR

**Keywords:** computer-aided design/computer-assisted manufacture (cad/cam), marginal fit, firing cycles, zrconia, cad cam, veneering porcelain

## Abstract

Introduction

Zirconia provides adequate mechanical strength to be used as a framework for all ceramic prostheses. Such prostheses must be covered with suitable porcelain to obtain good aesthetic results.

The aim

To study the effect of the firing cycle numbers of veneering ceramics (one cycle, two cycles, and three cycles) on the marginal fit of computer-aided design (CAD) and computer-aided manufacturing (CAM) zirconia bridges.

Materials and methods

The sample consisted of 30 full ceramic zirconia bridges, designed by CAD/CAM on a metal bridge model that was designed for this purpose. The sample was divided into three groups (N = 10); group A underwent a single firing cycle, group B underwent two cycles, and group C underwent three cycles. The copper model of the bridge was prepared to be a three-unit bridge, and the impressions of the metal models were taken to make zirconia cores. After that, the veneering ceramic layer and the micro-marginal gap were measured (in microns) on both the buccal and lingual surfaces of each bridge in the sample using the replica technique. A one-way ANOVA test was used to detect statistically significant differences between the groups.

Results

There were no significant differences between the studied groups in binary comparison; however, the arithmetic mean values of the marginal gap in group C were greater than all the studied groups.

Conclusion

Within the limitations of the current work, we found that increasing the number of firing cycles of zirconia cores affects the marginal fit; thus, it is recommended to follow the two firing cycle protocol for better adaptation of the CAD/CAM zirconia bridges.

## Introduction

Lower aesthetic results of metal-ceramic restorations led to the discovery of new restorative materials that have high physical and aesthetic properties [1]. This has prompted companies to develop new systems for metal-free ceramic prostheses, as these materials are characterized by chemical stability, biocompatibility, and optical properties similar to those of natural teeth and support the ceramic frame [2, 3], all of which highlight the important role of zirconia in such prostheses.

Zirconia has three main properties: high aesthetics, a higher corrosion resistance than metal, and a higher crack and fracture resistance than other types of porcelain [2]. Zirconia framework can be made by zirconia sintering or using computer-aided design or computer-aided manufacturing (CAD/CAM) systems, which is considered a simplified procedure that significantly reduces the cost of dental technical services, reduces the consumption of materials, increases productivity, and produces precise restorations while ensuring that the patient spends less time in the office [4].

The clinical success of full ceramic prostheses is related to many factors, but the marginal fit is the most important criterion [5]. Dissolution of the adhering cement, secondary caries, pulpitis, and gingivitis, as well as greater plaque accumulation with bone resorption, are observed in prostheses with poor marginal fit. In addition, a short margin may also cause caries at the finish line area [4, 5, 6].

Several methods were used to measure marginal fit, like visual examination with a dental probe, direct view using a light microscope or stereomicroscope, the cross-section technique, and the cement replica technique [7]. The replica technique depends on the injection of light silicon into the prosthesis, which is then placed on the die and removed after complete curing. Then, another light silicone of a contrasting color is injected into the prosthesis, after which the silicon layers are removed and cuts are made to obtain longitudinal sections through which the marginal or internal fit can be studied using a light or an electronic microscope [8]. Tooth type, cement space, veneering porcelain, and firing cycles are the most important factors affecting the accuracy of the marginal fit [9].

Zirconia provides adequate mechanical strength to be used as a framework for all ceramic prostheses [2]. Such prostheses must be covered with suitable porcelain to obtain good aesthetic results, and clinical studies have shown that veneering porcelain is the weakest part of such prostheses [10, 11].

The veneering process is available on a wide range of products that are used by the layering technique, the alternative injection method, or a combination of the two methods [12]. However, there are a number of factors that cause failure in the veneering of porcelain, of which chipping is the most frequent type of failure [13]. Hence, the purpose of this research was to study the effect of the number of firing cycles for veneering porcelain on the marginal fit of CAD/CAM zirconia bridges.

## Materials and methods

Sample description

The sample consisted of 30 full ceramic zirconia bridges, designed by CAD/CAM on a metal bridge model that was designed for this purpose. The sample was divided into three groups (N = 10): group A underwent a single firing cycle, group B underwent two firing cycles, and group C underwent three firing cycles.

**Figure 1:**
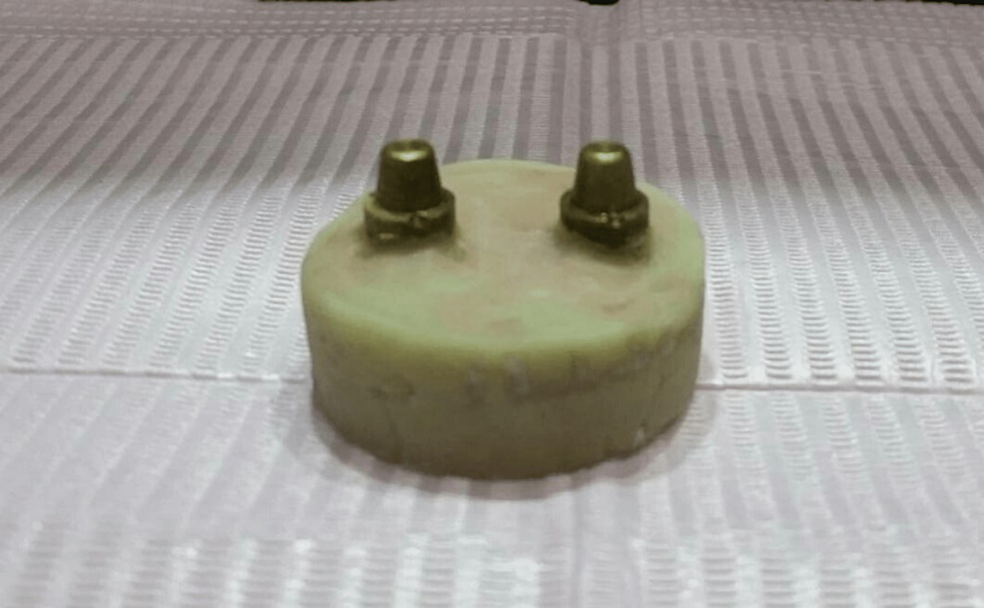
Standardized copper model fabrication

A copper metal model was made by an industrial lathe equipped with a protractor so that it would simulate the shape of prepared teeth in order to accurately adjust the angle of preparation and eliminate all other variables, such as the dimensions of the abutments and the width of the finish line that we could find if natural teeth were used. The dimensions of the model are as follows: (1) abutment diameter in the cervical area (8 mm), (2) abutment height (5 mm), (3) chamfer finish line (0.5 mm width), and (4) prepared walls angulation at 20° (Figure 1).

The impression for the copper model was taken by condensation silicone (c-silicon, Zetaplus Soft-Zhermack SpA, Italy), and it was then poured using die stone type IV (Velmix, Kerr Lab, Orange, CA, USA). A gypsum cast was sent to the dental lab to make zirconia cores as well as an acrylic base for the metal model.

Preparation of the zirconia cores

The gypsum cast was sprayed with a ScanMarker spray so that the scanning device can transfer data from the prepared teeth to the computer. It was then placed in the laser scanning device to obtain a three-dimensional image of the prepared model on the computer screen. The cement thickness was determined to be 30 microns, with a distance of 1 mm from the finish line, and the core thickness was 0.5 mm. Finally, the zirconia substructure was placed in a specific place within the carving device, where the design program would determine the places for carving the cores on it.

Firing veneering porcelain

The veneering porcelain (GC ZR-FS), which is compatible with the GC Initial Zr materials that have been used as zirconia core materials without any adverse reactions, was applied by mixing the ceramic powder with its liquid and brushing it on the zirconia core. It was applied 1 mm above the zirconia core margin to exclude any effect on the marginal fit. The veneering porcelain thickness was standardized at 1 mm for all the cores before firing using a thickness adjustment mold.

Using a porcelain oven (Multimat Cube, Dentsply Sirona, Charlotte, NC 28277, USA), a single firing cycle was performed on all samples to fire the first dentin layer. After that, group B underwent two firing cycles, of which the first one included firing the second dentin layer and the second one included adding a glaze layer on the porcelain surfaces. Meanwhile, group C underwent two correction powder-firing cycles. All previous steps were carried out according to the firing system of the used porcelain (Table 1).

**Table 1:** Porcelain firing system

	Preheating temperature	Drying time	Ralse of temperature	Vacuum	Final temperature	Holding time	Appearance
Shoulder firing	450°c	4 min	45°c/min	Yes	830°c	1 min	Shining
FM-firing	450°c	4 min	55°c/min	Yes	800°c	1 min	Slightly shining
First dentin firing	450°c	6 min	45°c/min	Yes	810°c	1 min	Slightly shining
Second dentin firing	450°c	6 min	45°c/min	Yes	800°c	1 min	Slightly shining
Glaze firing	480°c	2 min	45°c/min	ــــــــــــ	820°c	ـــــــ	Shining
Glaze firing with glaze powder	480°c	2 min	45°c/min	ــــــــــــ	790°c	1 min	Shining
Correction powder firing	450°c	4 min	45°c/min	Yes	690°c	1 min	Shining

Marginal fit assessment

**Figure 2:**
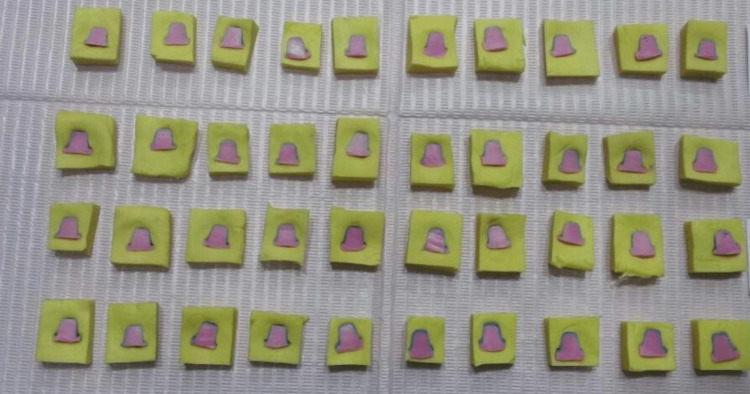
Injection of the additional low-viscosity silicone inside the c-silicone block

The marginal fit was assessed by measuring the marginal gap values (in microns) using the cement replica technique as follows: First, the stability of the bridges on the metal model was examined; following which, low-viscosity c-silicone was injected into each bridge. Then, the bridges were fixed to the metal model by finger pressure until the silicone had completed curing, upon which they were removed with the thin silicone layers. The bridges were then fixed on a high-viscosity c-silicone block, and low-viscosity addition silicone was injected into them by an electronic vibrator to ensure silicone reached and filled the smallest details of the c-silicone layers, thereby minimizing air bubbles within the molded silicone mass as much as possible (Figure 2).

**Figure 3:**
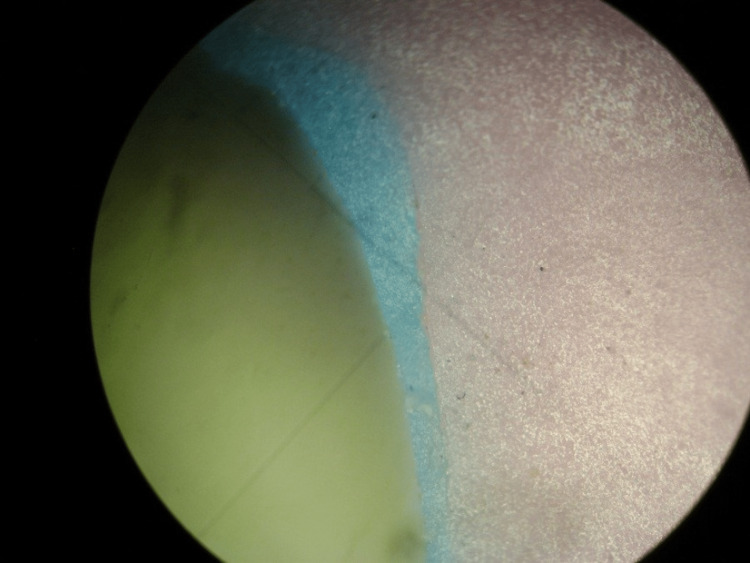
Sample measurement under the light microscope

After that, the molded silicone masses were separated from c-silicone blocks, trimmed, and divided into four equal pieces in the medial-distal direction by a fine scalpel to obtain four points for measuring the marginal fit. Each piece was kept in a special bag with the name of each section written on the bag. A light microscope with 10-fold magnification was used to measure these points. The measurement sample was fixed vertically to the microscope lens with a piece of synthetic silicone (Figure 3).

Statistical analysis

Data was collected and exported to Microsoft Excel 2013 (Microsoft Corporation). Then, statistical tests were conducted using Statistical Package for the Social Sciences (SPSS) version 19 (IBM SPSS Statistics, Armonk, NY, USA), with a significance level of 0.05. A one-way analysis of variance (ANOVA) test was used to study the difference between all studied groups in terms of the marginal gap value. A Bonferroni correction was used to study binary differences between the groups. The T-test for an independent sample was used to study the binary differences between buccal surface measurements and lingual surface measurements in each group.

## Results

Sample description

The sample consisted of 30 full ceramic zirconia bridges, which were distributed among the study groups as follows (Table 2).

**Table 2:** The sample distribution among the study groups

Groups	Number/Group	Percentage
Group A	10	33.3
Group B	10	33.3
Group C	10	33.3
Total	30	100

Comparison of the accuracy of marginal fit between the three groups

A one-way ANOVA test was used to study the differences in the mean values of the marginal gap (micron) between the three groups, according to the measurement site. It was shown that the p-value was less than 0.05, regardless of the measurement site, i.e., at the 95% confidence level, there are statistically significant differences in the mean of the marginal gap (micron) between at least two of the three groups. Therefore, to differentiate which groups are significantly different from the others, the binary comparison according to the measurement site was conducted using the Bonferroni correction (Tables 3, 4).

**Table 3:** Descriptive statistics of the marginal gap measurements in the study sample according to the measurement site. The studied variable is the marginal gap (micron). SD: standard deviation; S.Error: standard error; N: number A one-way ANOVA test was used to detect significant differences in the marginal gap values between the groups.

Measurement site	Groups	N.	Arithmetic mean	SD	S.Erorr	Max	Min
Buccal surface	Group A	10	114.20	24.72	7.82	80	150
	Group B	10	100.40	30.13	9.53	70	168
	Group C	10	120.50	14.03	4.44	90	140
Lingual surface	Group A	10	107.30	21.85	6.91	86	140
	Group B	10	100.10	31.50	9.96	60	156
	Group C	10	122.00	22.01	6.96	100	180
Both buccal and lingual surfaces	Group A	20	110.75	22.98	5.14	80	150
	Group B	20	100.25	30.00	6.71	60	168
	Group C	20	121.25	17.98	4.02	90	180

**Table 4:** One-way ANOVA test results F equals the mean square of the between-group divided by the mean square of the within-group; sig: statistical significance

Measurement site	F.ratio	p-value	Sig.
Buccal surface	6.997	0.001	Significant differences found
Lingual surface	9.647	0.000	Significant differences found
Both buccal and lingual surfaces	16.987	0.000	Significant differences found

Binary comparisons of the accuracy of the marginal fit between the study groups

A Bonferroni correction was used to study the binary comparison in the marginal gap measurements among groups, according to the measurement site. It was shown that the p-value is less than 0.05 when comparing the marginal gap measurements (in microns) between the bridge exposed to two firing cycles and the bridge exposed to three firing cycles on both the buccal and lingual surfaces. Therefore, at the level of confidence of 95%, there are statistically significant binary differences in the mean values of the marginal gaps (in microns) between these groups (Table 5).

As for the rest of the studied binary comparisons, it was noted that the p-value was higher than 0.05. Therefore, there were no statistically significant binary differences in the mean values of the marginal gaps (in microns) between groups.

**Table 5:** Bonferroni correction results Sig: statistical significance

Measurement site	Groups	Groups	Sig.
Buccal surface	Group A	Group B	No significant differences
		Group C	No significant differences
	Group B	Group C	No significant differences
Lingual surface	Group A	Group B	No significant differences
		Group C	No significant differences
	Group (B)	Group C	No significant differences
Both buccal and lingual surfaces	Group A	Group A	Significant differences found
		Group B	No significant differences
		Group C	No significant differences
	Group B	Group C	Significant differences found

The effect of the measurement site on the accuracy of the marginal fit for each group

The effect of the measurement site on the accuracy of the marginal fit for each group was measured separately. The T-test for independent samples was used to study binary differences in the mean values of the marginal gaps between the buccal surface measurement and lingual surface measurement according to the studied group (Table 6).

**Table 6:** Descriptive statistics of the measurements of marginal gaps on buccal and lingual surfaces according to the studied group N: number; SD: standard deviation.

Groups	Measurement site	N.	Arithmetic mean	SD
Group A	Buccal surface	10	114.20	24.72
	Lingual surface	10	107.30	21.85
Group B	Buccal surface	10	100.40	30.13
	Lingual surface	10	100.10	31.50
Group C	Buccal surface	10	120.50	14.03
	Lingual surface	10	122.00	22.01

It was shown that the p-value is much higher than 0.05. Therefore, at the 95% confidence level, there are no statistically significant differences in the mean values of the marginal gap measurements (in microns) between the measurements of the buccal and lingual surfaces, regardless of the number of firing cycles (Table 7).

**Table 7:** T-test for independent samples results T: T-test; Df: degree of freedom; Sig: statistical significance

Groups	T	Df	P.value	Sig.
Group A	0.661	18	0.517	No significant differences
Group B	0.022	18	0.983	No significant differences
Group C	-0.182	18	0.858	No significant differences

## Discussion

Lower aesthetic results of metal-ceramic restorations led to the discovery of new restorative materials that have high physical and aesthetic properties [1]. This prompted companies to develop new systems for metal-free ceramic prostheses, as these materials are characterized by chemical stability, biocompatibility, and optical properties similar to those of natural teeth and support the ceramic frame [2, 3]. So, this article aims to study the effect of the firing cycles’ number of veneering ceramics (one cycle, two cycles, and three cycles) on the marginal fit of CAD/CAM zirconia bridges.

A metal model was designed for this study in order to avoid the effect of different dimensions of the prepared tooth (the length and diameter of the abutment and the width of the preparation finishing line) on the results of the study. The method of obtaining the prepared metal abutments on an industrial lathe was used in several studies [14].

The convergence angle of 20° in the preparation was clinically acceptable, as clinical practitioners tend to prepare teeth with convergence angles ranging from 10° to 25° [15].

A quarter-ball notch was made on the outer edge of the semi-shoulder using a 1 mm ball bur (KOMET, Dentaltex, Madrid, Spain). This hole was intended to provide stability and correct the placement of the core on the abutment.

The thickness of the virtual cement layer (die spacer) was determined at 30 microns with a distance of 1 mm from the preparation finish line area. The thickness of this layer is an important factor in the internal and marginal fit, and Nakamura recommended adjusting the thickness of this layer by 30 microns to give a small margin gap [16]. The clinically acceptable value of the marginal gap is controversial in the medical literature. However, prostheses require a thickness of 30-80 mm for the adhesive cement layer [17].

The maximum clinically acceptable marginal gap value was determined at 120 µm [18, 19]. Full ceramic prostheses have values of marginal fit ranging from 17 µm to 161 µm using different manufacturing systems (casted glass ceramics, reinforced core systems, and machine milling), as the fit value is affected by the type of the used system [20].

A study model of metal was thus adopted to prevent any deformation, damage, or change in the margins while taking impressions. The applied forces on the crowns were unified even though they did not affect the differences in the marginal fit. Avoid modifying the inner surface of the crowns in order not to generate compressive stresses that harm the material structure and modify the occlusal surface, which may affect the marginal fit.

The marginal gap was measured from the farthest circumferential point of the outer edge of the prepared shoulder to the outermost circumferential point on the inner surface of the prosthesis. Moreover, all required measurements were made by the same person to avoid any influences due to individual factors.

The results of the study showed that when comparing the marginal gap values (in microns) between group C, which underwent three firing cycles, and the other studied groups, the p-value was less than 0.05. Thus, there were significant differences between them, as the arithmetic mean values of the marginal gap in group C were greater than all the other studied groups. This can be attributed to several factors, including the shrinkage of the veneering porcelain [21] or the effect of the firing process on the properties of the zirconia core, as firing heat is generally manifested in the structural change of the surface layer that may lead to some dimensional deformation [22] or the die distortion during the laboratory procedures [23].

The changes in the marginal fit can be attributed to the displacement of the crown margins towards the dental surfaces, which may lead to a tighter fit and then incomplete placement of the crown, thereby increasing the marginal gap [14].

The firing and cooling procedures produce residual tensile stress that occurs within the ceramic layer upon cooling under the glass transition stage, and in higher degrees, the ceramic gets rid of these stresses by deformation [24]. As a result of the absence of a linear relationship between the thermal expansion coefficient and the temperature change, the small differences in the thermal expansion coefficient will increase through the veneering porcelain layer and between the veneering layer and the zirconia core. Zirconia is a slow heat transfer material, so the faster the cooling, the greater the potential for stress [24].

DeHoff states that the difference in the coefficient of thermal expansion between the zirconia core and the veneering porcelain causes stresses to form when the restoration is cooled from the glass transition phase to room temperature, which results in the deformation of the restoration. Therefore, he suggested that the difference between the thermal expansion coefficient of veneering porcelain α and zirconia core α (Δα = α core -α veneering) should be within the range (-0.61 x 10-6.K-1 to +1.02 x10-6.K-1) [25].

The incompatibility of the coefficient of thermal expansion will put the porcelain veneer under pressure and reduce the possibility of cracks. In addition to the possibility of stresses occurring, which have a negative effect on the marginal fit of the zircon crowns, increasing the number of firing cycles leads to a change in this coefficient [26]. Even in the studies concerning metal-ceramic restorations, the marginal fit is affected by the number of firing cycles [27].

The results of our study agreed with a study conducted on the effect of firing ceramic on the marginal fit of two different types of zircon systems, Lava and Digident, as the results indicated that there were significant differences in the marginal fit before and after firing the veneering porcelain [28].

In addition, our results agreed with a new study that mentioned that multiple firing cycles can significantly improve the marginal fit of zirconia crowns [22].

On the other side, two studies that evaluated the marginal fit of four-unit zirconia bridges manufactured by three CAD/CAM systems before and after firing porcelain and glazing concluded that there was no effect of the processes of firing porcelain and glazing on the marginal fit of zirconia bridges [29, 30].

There were some limitations in this study, such as the study sample size and using only one firing system. This result may differ when using another heating temperature, and the internal fit may differ in the mouth, so clinical studies must be done to confirm this result.

## Conclusions

Based on the findings of this in vitro study, the following conclusions were drawn: increasing the number of firing cycles for veneering porcelain affects the marginal fit of CAD/CAM zirconia bridges, and the marginal fit was lower for three firing cycles compared to one and two firing cycles.
